# Structural and biochemical basis of the formation of isoaspartate in the complementarity-determining region of antibody 64M-5 Fab

**DOI:** 10.1038/s41598-019-54918-0

**Published:** 2019-12-06

**Authors:** Hideshi Yokoyama, Ryuta Mizutani, Shuji Noguchi, Naoki Hayashida

**Affiliations:** 1 Faculty of Pharmaceutical Sciences, Tokyo University of Science, 2641, Yamazaki, Noda, Chiba 278-8510 Japan; 2 Graduate School of Engineering, Tokai University, 4-1-1 Kitakaname, Hiratsuka, Kanagawa 259-1292 Japan; 3 Faculty of Pharmaceutical Sciences, Toho University, 2-2-1 Miyama, Funabashi, Chiba 274-8510 Japan; 4 Division of Molecular Gerontology and Anti-Ageing Medicine, Department of Biochemistry and Molecular Biology, Yamaguchi University Graduate School of Medicine, 1-1-1 Minami-Kogushi, Ube, Yamaguchi 755-8505 Japan

**Keywords:** Biochemistry, Structural biology

## Abstract

The formation of the isoaspartate (isoAsp) is one of spontaneous degradation processes of proteins, affecting their stability and activity. Here, we report for the first time the crystal structures of an antibody Fab that contains isoAsp in the complementarity-determining region (CDR), along with biochemical studies to detect isoAsp. By comparing the elution profiles of cation-exchange chromatography, it was clarified that the antibody 64M-5 Fab is converted from the normal form to isoAsp form spontaneously and time-dependently under physiological conditions. The isoAsp residue was identified with tryptic peptide mapping, N-terminal sequencing, and the protein isoaspartyl methyltransferase assay. Based on the fluorescence quenching method, the isoAsp form of 64M-5 Fab shows a one order of magnitude lower binding constant for its dinucleotide ligand dT(6–4)T than the normal form. According to the structure of the isoAsp form, the conformation of CDR L1 is changed from the normal form to isoAsp form; the loss of hydrogen bonds involving the Asn28L side-chain, and structural conversion of the β-turn from type I to type II’. The formation of isoAsp leads to a large displacement of the side chain of His27dL, and decreased electrostatic interactions with the phosphate group of dT(6–4)T. Such structural changes should be responsible for the lower affinity of the isoAsp form for dT(6–4)T than the normal form. These findings may provide insight into neurodegenerative diseases (NDDs) and related diseases caused by misfolded proteins.

## Introduction

**Figure 1:**
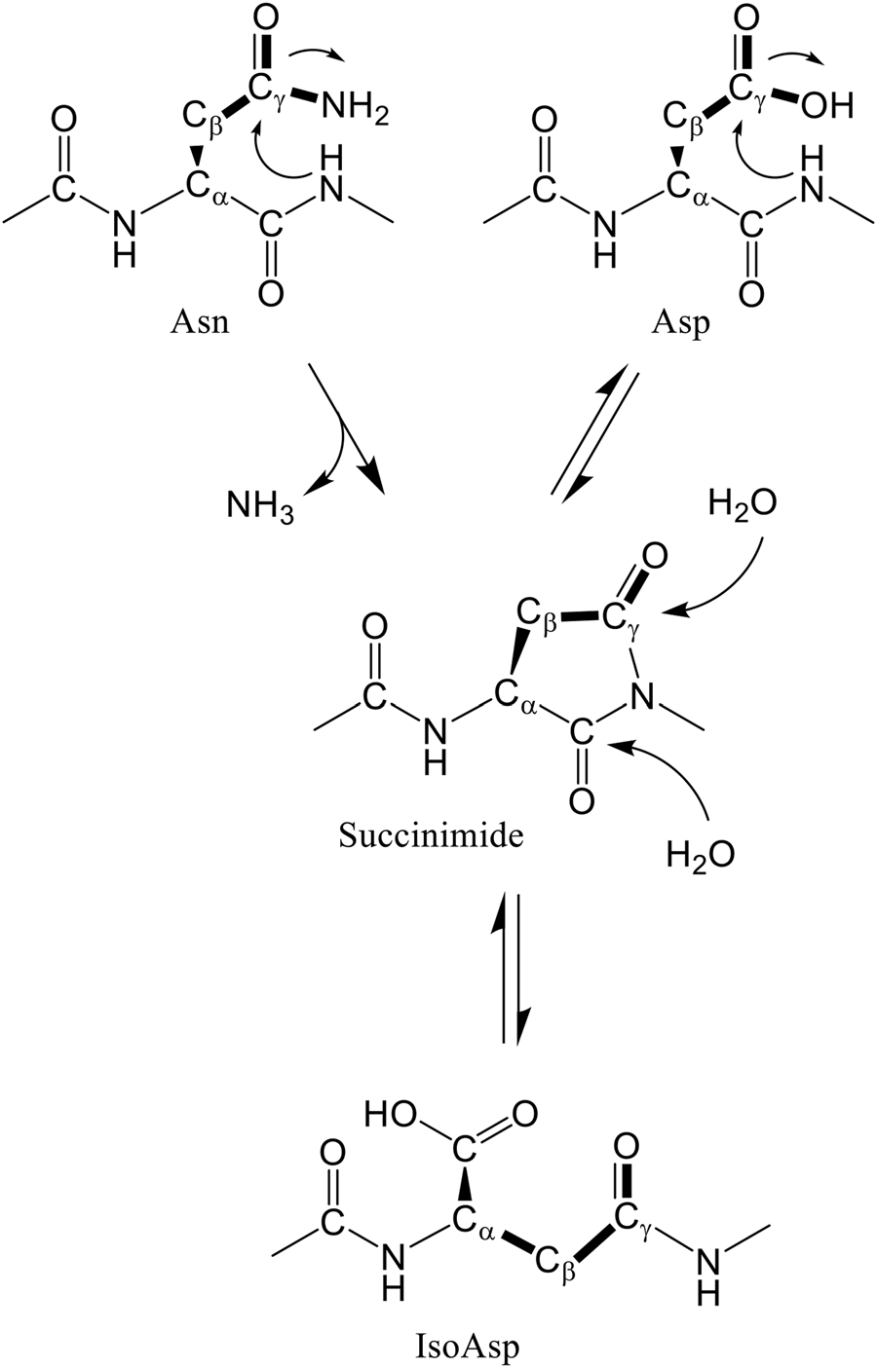
Deamidation and isomerization of asparagine^15^. Side-chain bonds of asparagine and aspartate are drawn as bold lines.

Proteins are subject to a variety of spontaneous degradation processes, such as oxidation, methylation, deamidation, and isomerization. The deamidation of asparagine and isomerization of aspartate proceed non-enzymatically under physiological conditions, and result in the formation of isoaspartate (isoAsp) via a five-membered cyclic succinimide intermediate^1,2^ (Fig. 1). These post-translational modifications occur in proteins, such as the β-amyloid protein from Alzheimer’s disease brains^3–5^, prion protein^6^, crystallins in the eye lenses^7^, histone H2B^8^, and Bcl-X_L_^9^. The formation of the major L-isoaspartyl products of these proteins can negatively affect their function. Hence, protein isoaspartyl methyltransferase (PIMT) converts isoaspartyl products to normal L-aspartyl residues^10,11^. PIMT-deficient mice accumulate isoaspartyl products and show significant growth retardation and fatal seizures^12,13^. Although a large number of proteins have been reported to contain isoAsp, three-dimensional structures of isoAsp-containing proteins have so far been determined in only a few cases; ribonucleases (RNases) U2 and A^14–17^, lysozyme^18^, MurA^19^, β-trypsin^20^, and Norovirus capsid protein VP1^21^. More structural information needs to be determined to understand how structural changes affect functions of proteins by forming isoAsp products.

In protein pharmaceuticals^22^, such as monoclonal antibodies^23–25^, the stability of proteins is likely to be altered by deamidation and isomerization of Asn at the surface of the proteins during prolonged storage. Although many therapeutic antibodies are in clinical use, there have been many reports about deamidation of Asn residues of complementarity-determining regions (CDRs)^26–31^. And such alterations decreases relative binding affinity for their ligands^26,28^. In many cases, LC-MS or LC-MS/MS methods are often used to detect isoAsp^27,29–31^, in combination with the PIMT assay, peptide mapping, N-terminal sequencing, and hydrophobic interaction chromatography. Recently, a high-throughput assay to monitor the deamidation of asparagine and isomerization of aspartate residues was reported^32^. However, there are no structural reports of isoAsp in CDRs of antibodies.

Here, we report structural and biochemical studies of isoAsp in CDRs of the monoclonal antibody 64M-5 Fab. The monoclonal antibodies 64M-5, 64M-2, and 64M-3 are highly specific for the DNA (6–4) photoproduct, and have been utilized in detection and quantification^33^. The major ultraviolet (UV)-irradiated DNA photoproducts at dipyrimidine sites include cyclobutane pyrimidine dimers and (6-4) photoproducts^34^. T(6-4)T photoproducts frequently cause T-to-C mutations at their 3′-sides during the replication of DNA and thus are mutagenic^35,36^. Our group previously determined the crystal structures of 64M-5 Fab and its complex with dT(6-4)T^37^, and also determined the crystal structure of a complex of 64M-5 Fab with a double-stranded DNA containing T(6-4)T^38^. During the preparation of 64M-5 Fab with cation-exchange chromatography, charge heterogeneity of Fab was observed as reported for other antibodies^24,25^. We analyzed the biochemical properties of this Fab heterogeneity, and determined the crystal structure of the isoAsp-containing 64M-5 Fab. To our knowledge, this is the first report to determine the structure of isoAsp in CDRs of monoclonal antibodies and discuss the structural basis of the decreased affinity of the antibodies due to the formation of isoAsp.

## Materials and Methods

### Preparation of the 64M-5 Fab

The antibody 64M-5 Fab was prepared as described previously^37^, according to the detailed preparation procedure for 64M-2 Fab^39^. In brief, the hybridoma cell line 64M-5 was grown in PFHM-II medium (Gibco) for about one month. After papain digestion and dialysis, the dialysate was loaded onto a Mono Q anion-exchange column (GE Healthcare), and the flow-through Fab fraction was loaded onto a Mono S cation-exchange column (GE Healthcare) equilibrated with 50 mM sodium acetate (pH 5.0), and eluted with a 0-400 mM NaCl gradient. Each of two major peaks (Frs. 1 and 2) was collected, and was subsequently applied to a Superdex 75 gel-filtration column (GE Healthcare) equilibrated with 50 mM Tris-HCl (pH 7.2) containing 150 mM NaCl for binding-affinity measurements and crystallization experiments.

### Tryptic peptide mapping

Each fraction of the Mono S eluate was lyophilized, then denatured and reduced using 7 M guanidine-HCl in Tris buffer (pH 8.6) and dithiothreitol (DTT), and subsequently carboxymethylated with iodoacetic acid. The sample was loaded onto a reversed-phase Phenyl-5PW column (Tosoh) equilibrated with 0.1% trifluoroacetic acid (TFA), and eluted with 0.1% TFA and 90% acetonitrile. The resulting light-chain (L-chain) fraction was lyophilized, and dissolved using 8 M urea in Tris buffer (pH 8.0) and kept for 15 min. The sample was diluted in a final concentration of 2 M urea, and digested using trypsin at an enzyme to protein ratio of 1:50 at 37 °C for 12 h. The reaction was quenched by adding 0.1 mM phenylmethylsulfonyl fluoride (PMSF). The reaction mixture was loaded onto a reversed-phase ODS-80Ts column (Tosoh) equilibrated with 0.1% TFA, and eluted with a gradient from 12–50% acetonitrile. The resulting peptide fragments were collected, and Matrix-Assisted Laser Desorption Ionization Time-of-Flight Mass Spectrometry (MALDI-TOF MS) analyses were performed (KOMPACT MALDI IV, Shimadzu) using α-cyano-4-hydroxycinnamic acid as a matrix.

### PIMT assay

The commercially available ISOQUANT isoaspartate detection kit (Promega) was used for the PIMT assay, and the reaction was conducted according to the manufacturer’s suggestions. Samples (100–150 pmol) were incubated at 30 °C for 30 min in the presence of PIMT and *S*-adenosyl methionine (SAM) in the reaction buffer. Then, stop solution was added to quench the reaction. The reaction mixture was loaded onto a reversed-phase ODS-80Ts column (Tosoh) equilibrated with 9 mM potassium phosphate (pH 6.2) and 10% methanol. The amount of isoAsp was determined by quantifying *S*-adenosyl homocysteine (SAH) eluted from the column.

### Binding-affinity measurement using fluorescence quenching

Binding affinities of dT(6-4)T to 64M-5 and 64M-3 Fabs were measured with the fluorescence quenching method reported by Azuma *et al*.^40^ with the Hitachi fluorescence spectrophotometer F-3010. The excitation and emission wavelengths were 280 and 340 nm, respectively, and the temperature was maintained at 25 °C. The titration was carried out by adding dT(6-4)T solution to Fab solution in 20 mM Tris-HCl (pH 7.2). The values for the binding constants were determined from non-linear least-squares fitting based on the equation describing ligand-protein binding^41^.

### Crystallization

Crystallization was performed at 20 °C by the sitting-drop vapor diffusion method. Crystals of 64M-5 Fab derived from Mono S Fr. 1 (isoAsp-form) were prepared using 8% (w/v) PEG3350, 8% (v/v) isopropanol, and 0.1 M sodium citrate (pH 5.6) as the reservoir solution and 20 mg/mL Fab in 50 mM Tris-HCl (pH 7.2) and 150 mM NaCl as the protein solution. Droplets were composed of equal volumes of the reservoir and protein solutions. Plate-shaped crystals grew to an approximate size of 0.4 × 0.2 × 0.05 mm. Crystals of the 64M-5 Fab Fr. 1 (isoAsp-form) – dT(6-4)T complex were obtained using 20 mg/mL (0.44 mM) Fab and 0.625 mM dT(6-4)T in 50 mM Tris-HCl (pH 7.2) and 150 mM NaCl as a protein solution, and 15% (w/v) PEG2000, 0.1 M ammonium sulfate, and 0.1 M sodium citrate (pH 3.5) as the reservoir solution. Rod-shaped crystals grew to an approximate size of 0.45 × 0.05 × 0.05 mm.

### X-ray data collection

Crystals were soaked in a cryoprotectant solution containing 20% glycerol, 16% PEG3350, 16% (v/v) isopropanol (unliganded form), or 18% glucose (dT(6-4)T-liganded form), and were flash-cooled in a nitrogen-gas stream at 105 K. Diffraction data were collected on an imaging plate detector (RAXIS IV, Rigaku) using graphite-monochromated Cu*K*α radiation from a rotating-anode generator (MAC science), as described previously^37,42^. Data sets were processed and scaled with the programs *DENZO* and *SCALEPACK*^43^.

### Structure determination and refinement

The crystal structures of the 64M-5 Fab (isoAsp-form) and 64M-5 Fab (isoAsp-form) – dT(6-4)T were determined by the molecular-replacement method and refined using *X-PLOR*^44^ and *CNS*^45^ with several cycles of manual model rebuilding using *TURBO-FRODO*^46^ and *Coot*^47^, as described previously^37^. In 64M-5 Fab (isoAsp-form), the structural model of Asn28L did not fit the electron density. The density was interpreted as that for isoaspartate, and an isoaspartate model from the β-L-aspartyl-L-alanine^48^ was finally built into the model. In 64M-5 Fab (isoAsp-form) – dT(6-4)T, the density of Asn28L is unclear, and so the model was not built into the density. The least-squares fittings of crystal structures were performed with *LSQKAB* in the *CCP4* suite^49^. All molecular figures were produced using *PyMOL* (http://www.pymol.org/).

## Results

### Conversion of Fr. 2 to Fr. 1 on cation-exchange column of 64M-5 Fab under physiological conditions

**Figure 2:**
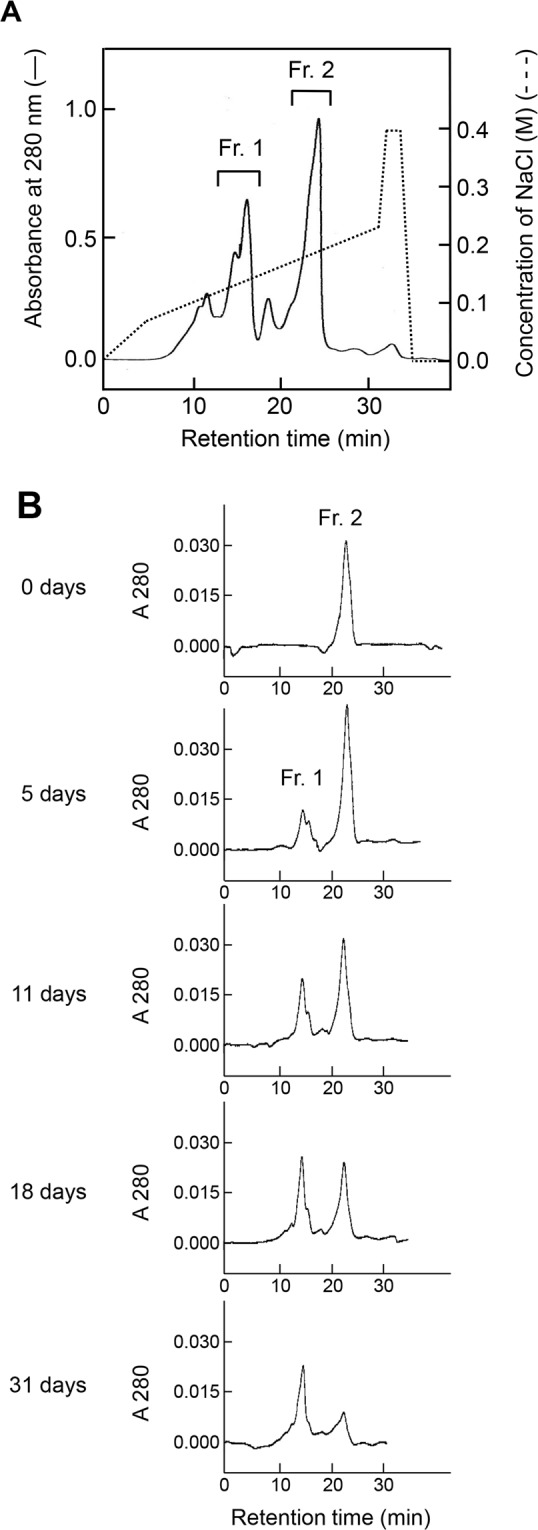
Charge heterogeneity and time-dependent change of 64M-5 Fab with cation-exchange chromatography. (**A**) An elution profile on a Mono S cation-exchange column. The solid line indicates absorbance at 280 nm of eluates, and the broken line indicates the ionic concentration. The Fr. 1 isoform was used for subsequent crystallographic analyses. (**B**) Elution profiles on a Mono S cation-exchange column after incubating the Fr. 2 isoform of 64M-5 Fab at pH 7.5 and 37 °C.

During the preparation of 64M-5 Fab using a Mono S cation-exchange column, charge heterogeneity of Fab was observed (Fig. 2A). The structures of 64M-5 Fab and its complex with the ligands were determined previously using the largest peak Fr. 2^37,38^. The purified Fr. 2 isoform was incubated under physiological conditions (0.1 M HEPES-NaOH pH 7.5, 37 °C) for up to a month, and elution profiles on a Mono S column were compared (Fig. 2B). The Fr. 1 isoform increased time-dependently in conjunction with the decrease of the Fr. 2 isoform. The relative ratio of the Fr. 1 yield was 24% at 5 days, 36% at 11 days, 46% at 18 days, and 62% at 31 days of incubation. Because Fr .1 eluted earlier than Fr. 2 on the cation-exchange column, the Fr. 1 isoform should be more acidic than Fr. 2. To check whether each fraction on the Mono S column contains isoAsp, a PIMT assay was performed (Supplementary Fig. S1). The assay identified isoAsp residues in the Fr. 1 isoform, but not in Fr. 2. These results indicate that the Fr. 2 isoform was non-enzymatically and time-dependently converted to the more acidic Fr. 1 isoform that contains isoAsp. It seems possible that peaks other than Frs. 1 and 2 correspond to an aspartate form produced via a succinimide intermediate (Fig. 1), and we cannot exclude the possibility that a shoulder peak of Fr. 1 may contain an aspartate form. It is reported that other isoforms, D-aspartate and D-isoaspartate, are slso produced via a succinimide intermediate^7^, and thus these isoforms may be included in other peaks.

### Detection of isoAsp28L

**Figure 3:**
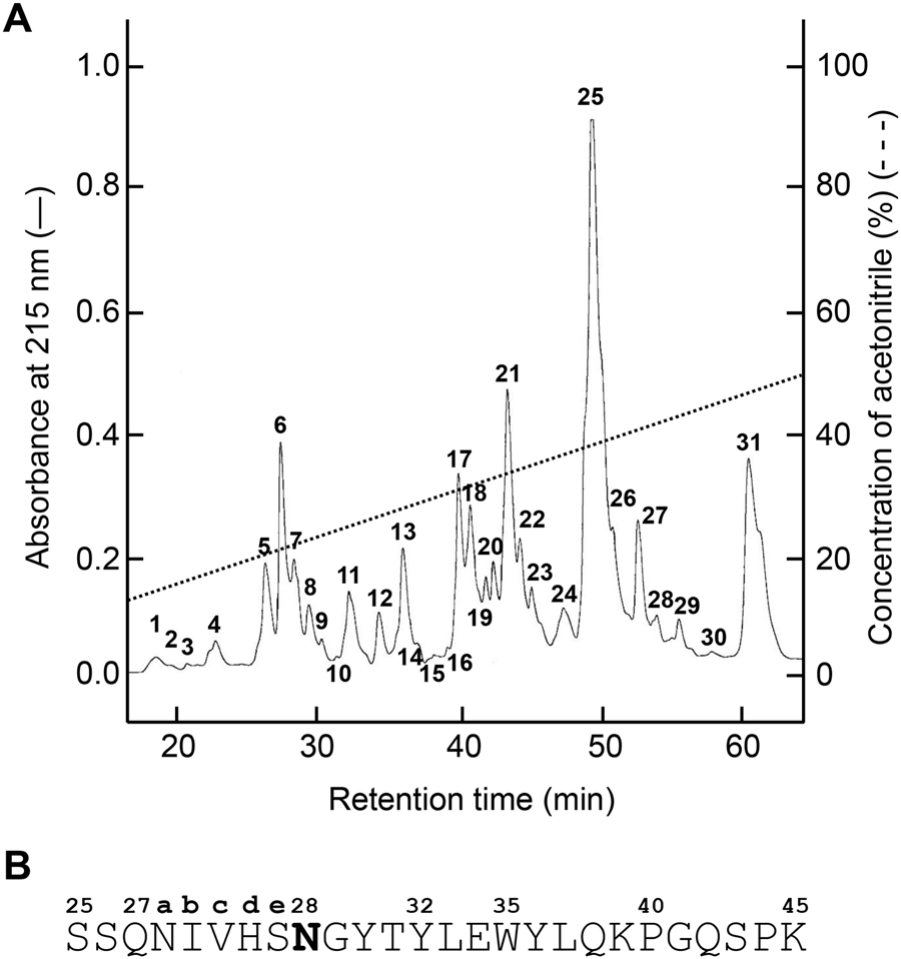
Separation of the tryptic digest of the L-chain fraction obtained from the Mono S Fr. 1 of 64M-5 Fab. (**A**) An elution profile on a reversed-phase ODS-80Ts column (Tosoh). (**B**) Amino acid sequence and numbering of the No. 25 peptide. Asn28L shown as a bold letter was identified as isoAsp.

To determine which residue is isoAsp, tryptic peptide mapping was performed. The Fr. 1 isoform of the Mono S eluate was lyophilized, denatured, and carboxymethylated, as described in Materials and Methods. The resultant L-chain fraction was isolated (Supplementary Fig. S2), digested using trypsin, and separated by reversed-phase chromatography (Fig. 3A). Most peaks of tryptic peptides were identified by MALDI TOF-MS (Table 1) based on the amino-acid sequence^51^. Among them, the largest peak (25) showed a mass of 3,029 that nearly corresponds to the calculated mass of the Ser25L–Lys45L peptide including 28 L residue (Table 1). The Ser25L–Lys45L peptide contains two Asn but no Asp residues (Fig. 3B). Edman degradation sequencing of this peak indicated that its 8 N-terminal residues are SSQNIVHS, which coincides with the N-terminus of the Ser25L–Lys45L peptide. However, the reaction was blocked at the next cycle after detecting the last Ser27eL, and the next Asn28L was not identified, although the precedent Asn27aL was detected. To prove the existence of isoAsp in this peptide, a PIMT assay was performed (Supplementary Fig. S3). The amount of isoAsp was determined to be 0.64 ± 0.10 pmol per 1.0 pmol peptide. The detection ratio was comparable to those of other studies reporting isoAsp-containing proteins^52,53^, indicating that the peptide contains one isoAsp, although we cannot exclude the possibility that a small amount of aspartate form produced via a succinimide intermediate may be included in the peptide. Formation of isoAsp is often observed in the Asn–Gly sequence^11,52^. Therefore, it was considered that isoAsp was formed at Asn28L–Gly29L. These residues are often observed in other antibodies^54,55^, and thus may be relevant to the function of antibodies due to being CDR residues. Although the Fr. 2 isoform of the Mono S eluate was analyzed with the same method, and almost the same elution profile of the tryptic peptides as in Fig. 3A was obtained, no isoAsp was detected for the No. 25 peptide from Fr. 2 (Supplementary Fig. S3). Since other Asn–Gly or Asp–Gly sequences are found in 64M-5; Asp151L—Gly152L and Asn157L−Gly158L (Table 1), isoAsp may be formed in these sequences.

**Table 1:** Mass spectrometric analysis of tryptic peptides deprived from the Mono S Fr. 1 and L-chain fraction of 64M-5 Fab.

Residue No.^a^	Sequences	Calculated mass	Observed mass	Peak No.^b^
1–24	DVLMTQTPLSLPVSLGDQASISCR	2,591	2,583	27
25–45	SSQNIVHSNGYTYLEWYLQKPGQSPK	3,026	3,029	25
46–54	LLIYTVSNR	1,079	1,080	11
55–74	FSGVPDRFSGSGSGTDFTLK	2,063	2,061	22, 23
75–90	ISRVEAEDLGVYYCFR	1,979	1,980	15
91–103	GSHVPTFGGGTK	1,145	1,147	12
104–107	LEIK	503	—	—
108–142	RADAAPTVSIFPPSSEQLTSGGASVVCFLNNFYPK	3,731	3,735	31
143–147	DINVK	589	—	—
148–155	WKIDGSER	991	992	13
156–169	QNGVLNSWTDQDSK	1,593	1,597	17, 18
170–188	DSTYSMSSTLTLTKDEYER	2,228	2,229	21
189–199	HNSYTCEATHK	1,349	1,353	6
200–207	TSTSPIVK	833	835	8
208–211	SFNR	524	524	5
212–214	NEC	423	—	—

^a^The residue numbering follows that of Kabat *et al*.^50^.

^b^Peak numbers correspond to those of Fig. 3.

### Binding constants of Frs. 1 and 2 of 64M-5 Fab

**Figure 4:**
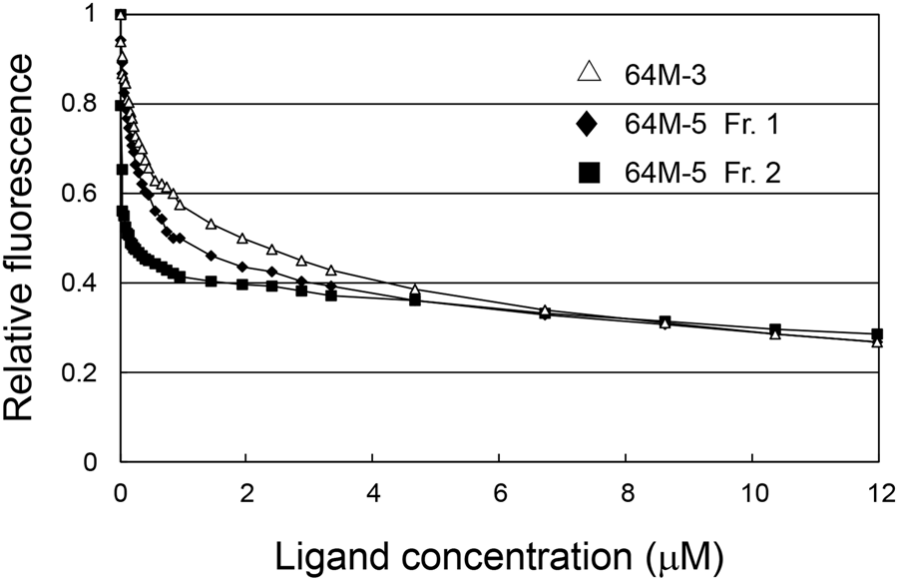
Fluorescence quenching of 64M-5 Fab isoforms. Changes in the relative fluorescence of 64M-5 Fab by adding the dT(6-4)T ligand are shown. The quenching profile of 64M-3 Fab is also shown.

Using the Fr. 1 and Fr. 2 isoforms from Mono S eluates, changes in the relative fluorescence were measured by adding the dT(6-4)T ligand (Fig. 4). The binding constants were determined by the fluorescence quenching and non-linear least-squares fitting^40,41^. The binding constant of 64M-5 Fr. 2 for dT(6-4)T was 9.9 ± 1.9 × 10^7^ M^−1^, and that of Fr. 1 was 5.2 ± 0.3 × 10^6^ M^−1^. The binding constant of 64M-3 Fab was also determined to be 2.4 ± 0.2 × 10^6^ M^−1^. In the previous surface plasmon resonance-based reports, the binding constant of 64M-5 for dT(6-4)T was at least one order of magnitude higher than that of 64M-3^56,57^, and thus the fluorescence quenching-based results shown here are considered to be plausible. The 64M-5 Fr. 1 isoform containing isoAsp28L shows a decreased binding constant, 1/20-fold of the Fr. 2 isoform. These results strongly indicate that the formation of isoAsp in 64M-5 Fab affects its binding affinity.

### Overall structures of the 64M-5 Fab Fr. 1 (IsoAsp form) and its complex with dT(6-4)T

**Figure 5:**
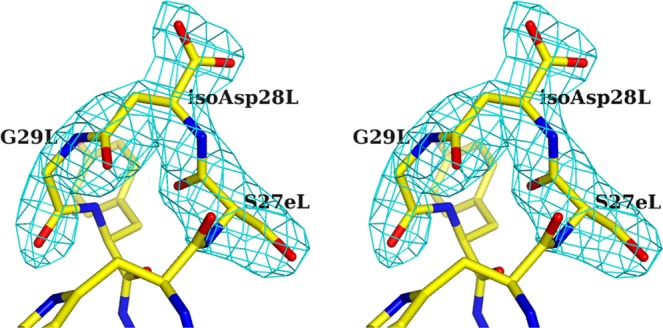
Stereo drawing of the *F*_o_ − *F*_c_ electron-density map and residues around isoAsp28L in the unliganded isoAsp form (2.47 Å resolution). The map was calculated based on phases from the model without residues Ser27eL, isoAsp28L, and Gly29L and is contoured at 4 σ. The isoAsp28L and surrounding residues are also shown as stick models.

To clarify the structural basis of the affinity decrease with isoAsp formation in 64M-5 Fab, crystal structures of 64M-5 Fab Fr. 1 and its complex with dT(6-4)T were determined. Data collection and refinement statistics are shown in Table 2. Hereafter, the Fr. 1 isoform is referred to as the isoAsp form. Structural models are observed with a clear electron density, except for residues in the heavy-chain constant domain (Ala130H, Gln133H, and Thr134H of the unliganded isoAsp form, and Ala129H, Ala130H, Gln133H, and Thr134H of the dT(6-4)T-liganded isoAsp form), which are disordered in most Fab structures^37,39,42,59^, and except for residue Asn28L of the dT(6-4)T-liganded isoAsp form. Figure 5 shows a clear electron density for the residues around isoAsp28L of the unliganded structure. The main-chain torsion angles of Fab were analyzed using RAMPAGE^58^, as shown in Table 2. His93L in the unliganded isoAsp form is in outlier regions. The residue was also in outlier regions in the previously reported structure^37^.

**Table 2:** Data collection and refinement statistics.

	64 M5 Fab (isoAsp-form)	64M5 Fab (isoAsp-form) ‒ dT(6-4)T
Space group	*C*222	*P*2_1_2_1_2_1_
**Cell dimensions**		
*a*, *b*, *c* (Å)	101.8, 150.5, 65.2	84.0, 102.9, 53.4
Resolution range (Å)	30.0–2.47 (2.55–2.47)^a^	30.0–2.70 (2.80–2.70)^a^
No. of observed reflections	87,861	46,785
No. of unique reflections	17,039 (1,271)	12,638 (967)
*R*_merge_ (*I*)^b^	0.071 (0.365)	0.090 (0.332)
Completeness	0.922 (0.734)	0.952 (0.750)
Average *I*/σ	18.8 (3.7)	14.5 (2.9)
*R*^c^/*R*_free_^d^	0.194/0.253	0.211/0.260
**No. of non-hydrogen atoms**		
Protein	3,345	3,318
Nucleotide	0	37
Water	241	93
**Average** ***B*** **factors (Å**^2^**)**		
Protein	32.6	36.4
Nucleotide	—	35.1
Water	35.1	26.5
**R.m.s. deviations from ideality**		
Bond lengths (Å)	0.007	0.004
Bond angles (°)	1.442	1.208
**Ramachandran plot**^**e**^ **(%)**		
Favored region	93.9	93.9
Allowed region	5.9	6.1
Outlier region	0.2	0.0

^a^Values in parentheses are for the highest-resolution shell.

^b^*R*_merge_ (*I*) = Σ_*hkl*_Σ_*j*_ | *I*_*j*_(*hkl*) − < *I* (*hkl*) > |/Σ_*hkl*_Σ_*j*_*I*_*j*_(*hkl*), where *I*_*j*_(_*hkl*_) is the intensity of an individual reflection and <*I* (*hkl*) >is the mean intensity of that reflection.

^c^*R* = Σ_*hkl*_ | |*F*_obs_| − |*F*_calc_| |/Σ_*hkl*_ |*F*_obs_|, where |*F*_obs_| and |*F*_calc_| are the observed and calculated structure factor amplitudes, respectively.

^d^*R*_free_ is calculated for 10% of the reflections randomly excluded from refinement.

^e^Values were calculated with RAMPAGE^58^.

The root-mean-square deviation (r.m.s.d.) for the main-chain atoms of the variable regions (V_L_ and V_H_) between the unliganded and dT(6-4)T-liganded isoAsp forms is 0.99 Å. The r.m.s.d. between unliganded isoAsp (Fr. 1) and normal (Fr. 2) forms^37^ is 0.82 Å, and that between dT(6-4)T-liganded structures of the isoAsp and normal forms^37^ is 0.72 Å. The structures of the variable regions of these Fabs are similar, and thus we hereafter compare the CDRs and ligand-binding sites.

### Structure of CDR L1 of the 64M-5 Fab IsoAsp form

**Figure 6:**
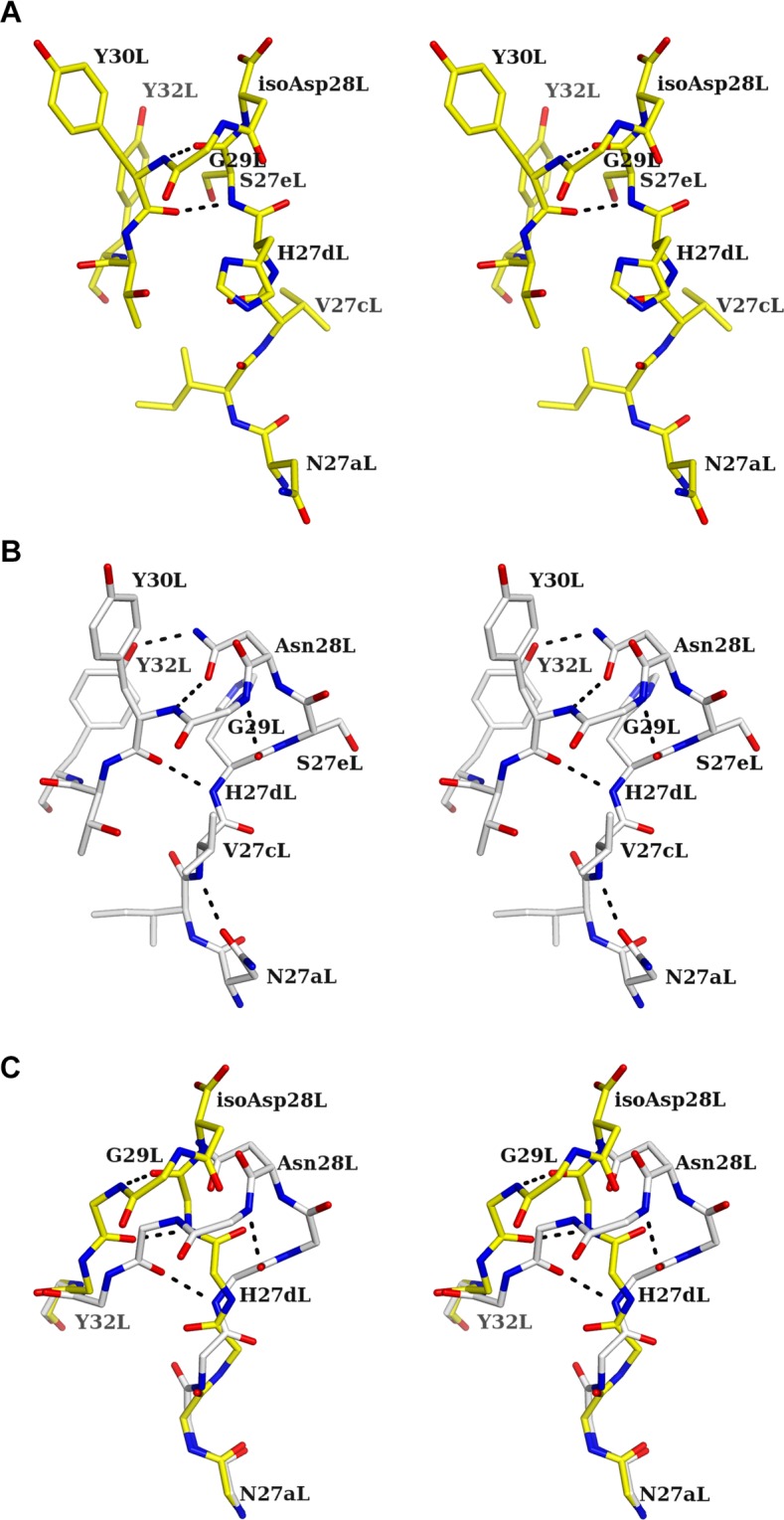
Stereo drawing of CDR L1 residues of the unliganded isoAsp and normal forms. Hydrogen bonds are shown as broken lines. (**A**) The structure of the unliganded isoAsp form of 64M-5 Fab is colored yellow. (**B**) The structure of the unliganded normal form of 64M-5 Fab is colored grey. (**C**) Superposition of the unliganded isoAsp (yellow) and normal (grey) forms. Side-chain atoms except for isoAsp28L or Asn28L have been omitted for clarity.

To elucidate the structural changes of CDR residues accompanied by the formation of isoAsp, CDR residues of unliganded isoAsp were compared with those of normal forms^37^ by superposing the main-chain variable regions of Fab. Major differences are observed for CDR L1, with an r.m.s.d. for the main-chain atoms of L1 (residues 24L-34L) of 1.55 Å, whereas the r.m.s.d.s for the main-chain atoms of L2 (residues 50L-56L), L3 (residues 89L-97L), H1 (residues 31H-35H), H2 (residues 50H-65H), and H3 (residues 95H-102H) are 0.31, 0.66, 0.18, 0.24, and 0.21 Å, respectively. The conformation of CDR L1 was completely rearranged in the isoAsp form compared with the normal form. In CDR L1 of the isoAsp form, main-chain hydrogen bonds are observed only between Ser27eL N and Tyr30L O and between Ser27eL O and Tyr30L N (Fig. 6A). In the normal form, these hydrogen bonds were not observed; instead, a total of five hydrogen bonds are formed in CDR L1. Those are main-chain hydrogen bonds between His27dL N and Tyr 30 L O and between His27dL O and Gly29L N and side-chain hydrogen bonds between Asn28L Oδ1 and Tyr30L N, between Asn28L Nδ2 and Tyr32L Oη, and between Asn27aL Oδ1 and Val27cL N (Fig. 6B). The two main-chain hydrogen bonds in the normal form are typical for the type 4 canonical conformation of CDR L1^37,60^, though they were not retained in the isoAsp form. A structural change from the normal to isoAsp form would cause the loss of hydrogen bonds; in particular, loss of two hydrogen bonds involved in the side chain of Asn28L should cause the rearrangement of the main-chain conformation of L1 (Fig. 6C). The tip of L1 (around residue 28 L) of the normal form adopts a type I β-turn, while that of the isoAsp form adopts a type II’ β-turn (β-turn classification is from Rose *et al*.^61^). By comparing the inter-atomic distances between the CDR L1 Cα atoms of isoAsp and normal forms, Ser27eL Cα shows the largest displacement (5.7 Å).

In the case of the unliganded structure, the average *B*-factor of the residues from Asn27aL to Tyr32L of the isoAsp form is 48.0 Å^2^ (the value of all protein residues is 32.6 Å^2^), whereas that of the normal form is 23.1 Å^2^ (all protein, 36.7 Å^2^). In the case of the dT(6-4)T-liganded structure, that of the isoAsp form is 50.1 Å^2^ (all protein, 36.4 Å^2^), whereas that of the normal form is 22.9 Å^2^ (all protein, 28.0 Å^2^). The high *B*-factor of the residues surrounding isoAsp28L would correlate with the loss of hydrogen bonds.

### Conformational differences in the ligand-binding site induced by the formation of isoAsp

**Figure 7:**
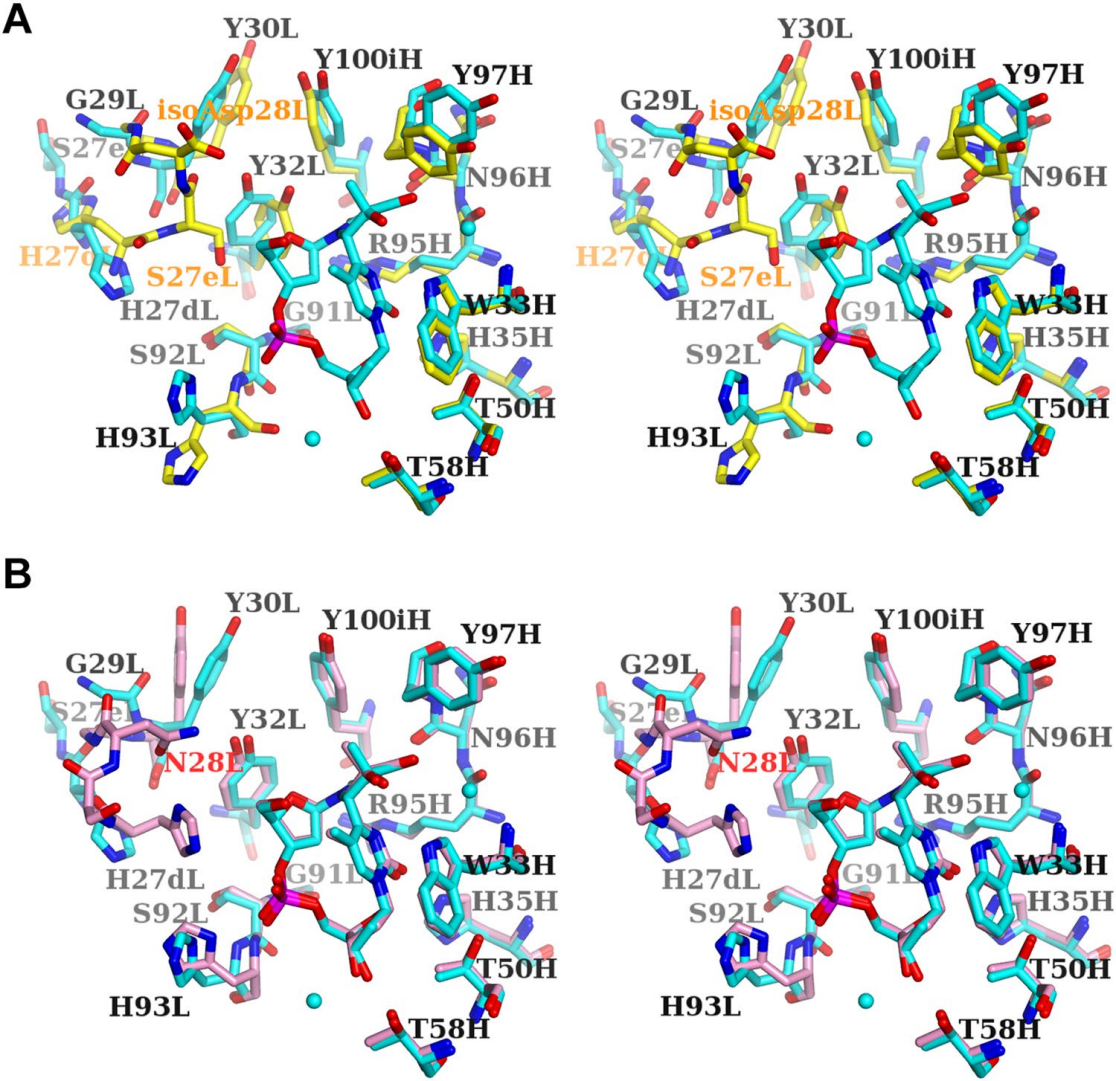
Comparison of the ligand-binding sites of 64M-5 Fab (stereo views). (**A**) The structure of the unliganded isoAsp form is colored yellow, and that of dT(6-4)T-liganded isoAsp form is colored cyan. Water molecules in the dT(6-4)T-liganded form are shown as spheres. Residues of the dT(6-4)T-liganded form are labeled in black, and those of the unliganded form are orange. In the dT(6-4)T-liganded isoAsp form, isoAsp28L is not included in the model. (**B**) Structures of the dT(6-4)T-liganded isoAsp (cyan) and normal (pink) forms. The residue Asn28L of the normal form is labeled in red.

To elucidate the structural basis of the decreased affinity due to the formation of isoAsp, we compared the structures of the ligand-binding sites of 64M-5 Fabs. When comparing the dT(6-4)T-liganded and unliganded isoAsp forms, CDR L1 and L3 residues show large differences (Fig. 7A). The side chain of His93L in L3 of the dT(6-4)T-liganded structure rotates by approximately 100° in χ1 (the torsion angle between Cα and Cβ) and by approximately 130° in χ2 (the torsion angle between Cβ and Cγ), and thus the Nδ1 atom shifts by 4.3 Å in the direction towards the phosphate group of dT(6-4)T. Similar conformational change is also observed in the dT(6-4)T-liganded and unliganded normal forms^37^. Characteristic conformational changes are observed in L1. As described above, the L1 residues from Asn27aL to Tyr32L in the isoAsp forms show high *B*-factors, and exhibit large displacement upon the binding of dT(6-4)T. The inter-atomic distance of His27dL Cα between the dT(6-4)T-liganded and unliganded isoAsp forms is relatively large (3.0 Å), resulting in pointing the side chain of His27dL in the opposite direction. The side chain of Tyr32L rotates by approximately 90° in its χ^2^ to accommodate the dT(6-4)T in the binding pocket.

When comparing the dT(6-4)T-liganded isoAsp form with the dT(6-4)T-liganded normal form^37^, most CDR residues except L1 and L3 show similar conformations (Fig. 7B). The side chain of His93L in L3 of the isoAsp form rotates and shifts slightly to point its Nδ1 atom away from the phosphate group of dT(6-4)T. His27dL in L1 shows a large difference. The His27dL Nε2 of the isoAsp form shifts by 5.0 Å in the direction opposite to dT(6-4)T. Therefore, the electrostatic interaction of His27dL with the phosphate group of dT(6-4)T is diminished by the formation of isoAsp, which would decrease the binding constant of Fab toward dT(6-4)T.

## Discussion

### Regional conformational changes induced by the formation of isoAsp

This is the first report to elucidate the structure of antibody Fab that contains isoAsp in CDR. By the methods of tryptic peptide mapping, N-terminal sequencing, MALDI TOF-MS, and the PIMT assay, it has become evident that Asn28L in CDR L1 of 64M-5 Fab is non-enzymatically converted to isoAsp during the prolonged culture of hybridoma cells under physiological conditions. The conformation of CDR L1 of the isoAsp-containing 64M-5 Fab is largely changed by the formation of isoAsp. Especially, the conformational change in His27dL should be responsible for the decrease in the binding constant for dT(6-4)T. Hence, the formation of isoAsp induces regional conformational changes and affects the biological activity of the antibody.

In the crystal structure of RNase U2 from *Ustilago sphaero-gena* containing isoAsp45^14^, the conversion of Asp45 to isoAsp induces changes in the main- and side-chain conformations and spatial arrangement of residues Tyr44-Asp50. These changes also alter the directions of the side chains of residues Glu46, Ser48, Glu49, and Asp50, and induce the structural change of the recognition site of RNase U2. In hen egg lysozyme, the conversion of Asp101 to isoAsp induces changes in its spatial arrangement, which results in the decreased binding constant of lysozyme by the formation of isoAsp^18^. In anti-IgE antibody E25, known as an anti-allergic therapeutic drug, the formation of isoAsp in CDR L1 and the reduction of affinity were reported^26^. Our study may help to explain the characteristic changes in the therapeutic antibody E25.

Accompanied by the conversion of Asn28L to isoAsp in 64M-5 Fab, hydrogen-bond patterns of CDR L1 are also converted. Asn28L and Gly29L are the residues forming a succinimide intermediate (Fig. 1). Thus, it is thought that three hydrogen bonds formed by Asn28L and Gly29L (Fig. 6B) were broken as soon as the succinimide intermediate was formed. The deletion of hydrogen bonds might induce the destabilization of the L1 loop and also induce the conformational changes of the residues. As shown in Fig. 1, the initial step in the isomerization of Asn (or Asp) occurs via nucleophilic attack of subsequent Gly N atom on Asn (or Asp) Cγ atom. In the structure of the unliganded normal form (Fig. 6B), a distance between Gly29L N and Asn28L Cγ is 3.6 Å. In other Asn–Gly or Asp–Gly sequences of 64M-5 (Table 1), these distances are relatively long; 5.0 Å for Asp151L–Gly152L and 4.6 Å for Asn157L–Gly158L. That is because side chains of Asp151L and Asn157L are in extended conformations to interact with other residues. Therefore, Asn28L should be most susceptible for the isomerization via a succinimide intermediate compared with the other Asp/Asn-Gly pairs.

### Formation of isoAsp in neurodegenerative diseases (NDDs)

There has been an increase in reports of proteins containing isoAsp *in vivo*. Most of these reports showed that the formation of isoAsp has adverse biological effects and is relevant to NDDs and aging. In β-amyloid peptides detected in Alzheimer’s disease patients’ brains, the formations of isoAsp at Asp1 and Asp7 were reported^3^. β-Amyloid is a peptide composed of various numbers of residues, mainly 40 (Aβ(1-40)) and 42 (Aβ(1–42)). In addition, Aβ(1–43) has also been recognized as a toxic peptide in the last two decades^62–65^. These peptides are produced by an abnormal cleavage from the amyloid precursor protein (APP) by β-secretase and γ-secretase. α-Secretase produces non-toxic amyloid peptide. However, these enzymes also cleave Notch signaling protein and their specificity is not high. Therefore, various lengths of amyloid peptides can be produced from APP.

An amyloid cascade hypothesis of Alzheimer’s disease has been proposed based on the propensity for oligomerization and amyloid fibril formation^66,67^. In addition, a hypothesis based on evidence from the formation of isoAsp in β-amyloid peptide has also been suggested^4^. β-Amyloid peptide has three Asp residues, and the conversion of Asp1 and Asp7 to isoAsp was discovered. The generation of two isoAsp residues changes the usual β-turn to an unusual type II’ β-turn. The type II’ β-turn is common in β-sheet structures, and β-sheet structures make non-specific protein-protein interactions possible and have a propensity to form fibrils. Formation of isoAsp might have an important role in the generation of protein aggregates (inclusion bodies) found in neurons of NDD patients’ brains through the mechanism described here.

During this period, the disorders ascribable to unusual protein conformations have been called NDDs. To our knowledge, the conversions of structures from an α-helix to β-sheet^68^ are involved in the specific proteins causing NDDs. If an Asp residue in an intramolecular α-helix undergoes isoAsp formation, the corresponding peptide stretch can be converted to a β-strand, and a β-sheet structure can be formed between different domains of other different proteins and facilitates non-specific protein-protein interactions. Therefore, isoAsp formation can cause abnormal protein aggregation and unusual protein recognition. From this point of view, a β-sheet breaker peptide was designed as a therapeutic drug and proved to be effective against Alzheimer’s and prion diseases^69,70^. In CDR L1 of 64M-5 Fab, the conversion of Asn28L to isoAsp changed the conformation of L1 from the type I to type II’ β-turn (Fig. 6). Induction of the type II’ β-turn leads to a β-sheet conformation, which enables non-specific protein-protein interaction and fibril formation. If a similar type II’ β-turn induction is discovered in proteins causing NDDs, conformational changes induced by the formation of isoAsp must be considered to have some relationship with the onset and progression of NDDs.

## Supplementary information

Supplementary information

## Data Availability

The atomic coordinates and structure factors have been deposited in the Protein Data Bank Japan (PDBj) with the accession codes 6KDH (in an unliganded isoAsp-form) and 6KDI (in a dT(6-4)T-liganded isoAsp-form).
