# Supplementary material for: Structural and biochemical basis of the formation of isoaspartate in the complementarity-determining region of antibody 64M-5 Fab
Source: Sci Rep. 2019 Dec 6;9:18494. doi: 10.1038/s41598-019-54918-0 (PMC6898713; doi:10.1038/s41598-019-54918-0)
Supplement: Supplementary file 1 — Supplementary information [file 41598_2019_54918_MOESM1_ESM.pdf]

Supplementary information

Structural and biochemical basis of the formation of isoaspartate in the complementarity-determining region of antibody 64M-5 Fab

Hideshi Yokoyama[1], Ryuta Mizutani[2], Shuji Noguchi[3], and Naoki Hayashida[4,*]

[1] Faculty of Pharmaceutical Sciences, Tokyo University of Science, 2641 Yamazaki, Noda, Chiba 278-8510, Japan.

[2] Graduate School of Engineering, Tokai University, 4-1-1 Kitakaname, Hiratsuka, Kanagawa 259-1292, Japan.

[3] Faculty of Pharmaceutical Sciences, Toho University, 2-2-1 Miyama, Funabashi, Chiba 274-8510, Japan.

[4] Division of Molecular Gerontology and Anti-Ageing Medicine, Department of Biochemistry and Molecular Biology, Yamaguchi University Graduate School of Medicine, 1-1-1 Minami-Kogushi, Ube, Yamaguchi 755-8505, Japan.

*Corresponding author: Naoki Hayashida

Division of Molecular Gerontology and Anti-Ageing Medicine, Department of Biochemistry and Molecular Biology, Yamaguchi University Graduate School of Medicine, 1-1-1 Minami-Kogushi, Ube, Yamaguchi 755-8505, Japan

E-mail: hayasida@yamaguchi-u.ac.jp; Phone: +81-836-22-2359; Fax: +81-836-22-2315

Contents

Figure S1. Detection of isoAsp using each fraction on the Mono S column.

Figure S2. Isolation of the light-chain (L-chain) fraction.

Figure S3. Detection of isoAsp using each tryptic peptide No. 25.

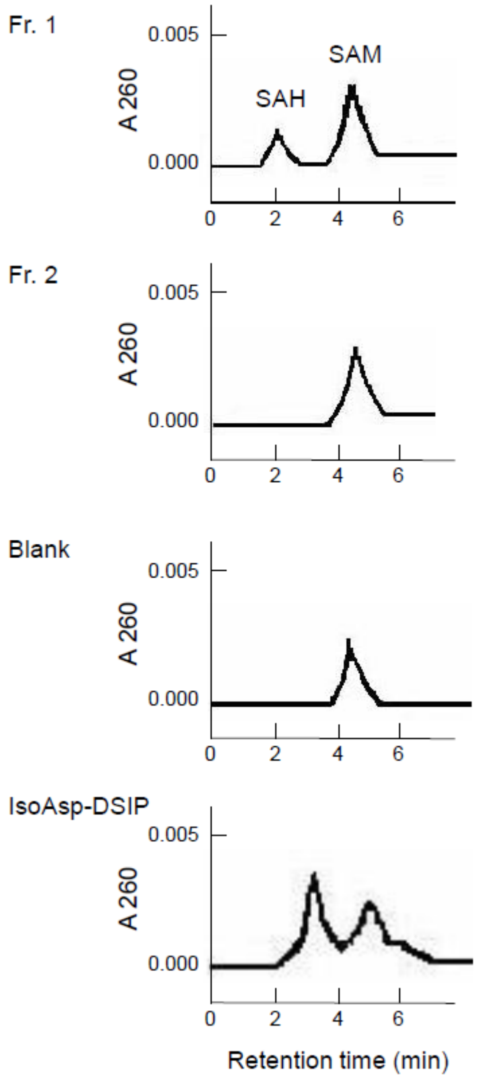

Figure S1. Detection of isoAsp using each fraction on the Mono S column. Elution profiles on a reversed-phase ODS-80Ts column (Tosoh) after the PIMT reaction using the Mono S eluates of 64M-5 Fab were compared. Fr. 1 and Fr. 2 are Mono S eluates. Blank is a buffer solution of the Mono S column as a negative control. IsoAsp-DSIP is a positive control of the ISOQUANT isoaspartate detection kit (Promega). After PIMT reaction, the substrate *S*-adenosyl methionine (SAM) and product *S*-adenosyl homocysteine (SAH) were detected using absorbance at 260 nm. These results indicate that Fr. 1 isoform contained isoAsp, but Fr. 2 did not.

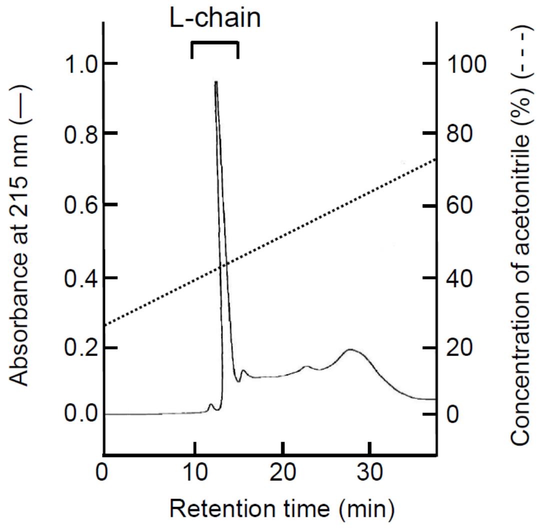

Figure S2. Isolation of the light-chain (L-chain) fraction. The Fr. 1 isoform of the Mono S eluate was lyophilized, denatured, and carboxymethylated as described in the "Tryptic peptide mapping" section of Materials and Methods of the main text. The resultant sample was applied to a reversed-phase Phenyl-5PW column (Tosoh) to isolate the L-chain fraction indicated with a label in the elution profile.

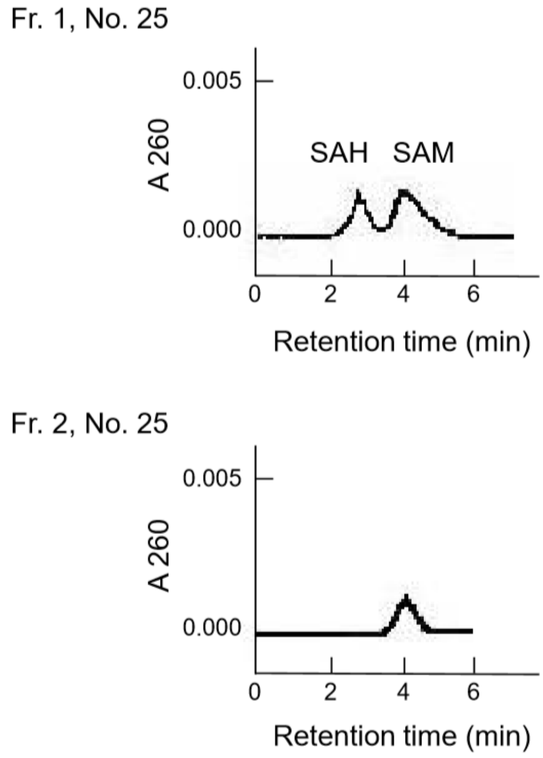

Figure S3. Detection of isoAsp using each tryptic peptide No. 25. Tryptic peptide No. 25 (Figure 3 in the main text) of the L-chain fraction isolated from the denatured and carboxymethylated Fr. 1 or Fr. 2 isoform of the Mono S eluate of the 64M-5 Fab was subject to PIMT reaction. Elution profiles on a reversed-phase ODS-80Ts column (Tosoh) after the PIMT reaction were compared. These results indicate that peptide No. 25 from the Fr. 1 isoform contained isoAsp, but that from Fr. 2 did not.
